# Real-time RT-PCR for Venezuelan equine encephalitis complex, Madariaga, and Eastern equine encephalitis viruses: application in human and mosquito public health surveillance in Panama

**DOI:** 10.1128/jcm.00152-23

**Published:** 2023-11-20

**Authors:** Jean-Paul Carrera, Dimelza Araúz, Alejandra Rojas, Fátima Cardozo, Victoria Stittleburg, Ingra Morales Claro, Josefrancisco Galue, Carlos Lezcano-Coba, Filipe Romero Rebello Moreira, Luis Felipe -Rivera, Maria Chen-Germán, Brechla Moreno, Zeuz Capitan-Barrios, Sandra López-Vergès, Juan Miguel Pascale, Ester C. Sabino, Anayansi Valderrama, Kathryn A. Hanley, Christl A. Donnelly, Nikos Vasilakis, Nuno R. Faria, Jesse J. Waggoner

**Affiliations:** 1 Department of Biology, University of Oxford, Oxford, United Kingdom; 2 Pandemic Sciences Institute, Nuffield Department of Medicine, University of Oxford, Oxford, United Kingdom; 3 Department of Research in Virology and Biotechnology, Gorgas Memorial Institute of Health Studies, Panama City, Panama; 4 Viral Emerging Disease Dynamics Group, Gorgas Memorial Institute of Health Studies, Panama City, Panama; 5 Departamento de Producción, Instituto de Investigaciones en Ciencias de la Salud, Universidad Nacional de Asunción, San Lorenzo, Paraguay; 6 Division of Infectious Diseases, Department of Medicine, Emory University, Atlanta, Georgia, USA; 7 Instituto de Medicina Tropical, Faculdade de Medicina da Universidade de São Paulo, São Paulo, Brazil; 8 MRC Centre for Global Infectious Disease Analysis (MRC-GIDA), Department of Infectious Disease Epidemiology, Imperial College London, London, United Kingdom; 9 Departamento de Genética, Universidade Federal do Rio de Janeiro, Rio de Janeiro, Brazil; 10 Departamento de Microbiología y Parasitología, Facultad de Ciencias Naturales, Exactas y Tecnología, Universidad de Panamá, Ciudad de Panamá, Panama; 11 Clinical of Tropical Diseases and Research Unit, Gorgas Memorial Institute of Health Studies, Panama City, Panama; 12 Department of Medical Entomology, Gorgas Memorial Institute of Health Studies, Panama City, Panama; 13 Department of Biology, New Mexico State University, Las Cruces, New Mexico, USA; 14 Department of Statistics, University of Oxford, Oxford, United Kingdom; 15 Department of Pathology, The University of Texas Medical Branch, Galveston, Texas, USA; 16 Department of Preventive Medicine and Population Health, The University of Texas Medical Branch, Galveston, Texas, USA; 17 Center for Biodefense and Emerging Infectious Diseases, The University of Texas Medical Branch, Galveston, Texas, USA; 18 Center for Vector-Borne and Zoonotic Diseases, The University of Texas Medical Branch, Galveston, Texas, USA; 19 Center for Tropical Diseases, The University of Texas Medical Branch, Galveston, Texas, USA; 20 Institute for Human Infection and Immunity, The University of Texas Medical Branch, Galveston, Texas, USA; 21 Department of Global Health, Rollins School of Public Health, Emory University, Atlanta, Georgia, USA; Mayo Clinic, Rochester, Minnesota, USA

**Keywords:** Venezuelan equine encephalitis, Madariaga virus, Eastern equine encephalitis virus, alphavirus, rRT-PCR

## Abstract

Eastern equine encephalitis virus (EEEV), Madariaga virus (MADV), and Venezuelan equine encephalitis virus complex (VEEV) are New World alphaviruses transmitted by mosquitoes. They cause febrile and sometimes severe neurological diseases in human and equine hosts. Detecting them during the acute phase is hindered by non-specific symptoms and limited diagnostic tools. We designed and clinically assessed real-time reverse transcription polymerase chain reaction assays (rRT-PCRs) for VEEV complex, MADV, and EEEV using whole-genome sequences. Validation involved 15 retrospective serum samples from 2015 to 2017 outbreaks, 150 mosquito pools from 2015, and 118 prospective samples from 2021 to 2022 surveillance in Panama. The rRT-PCRs detected VEEV complex RNA in 10 samples (66.7%) from outbreaks, with one having both VEEV complex and MADV RNAs. VEEV complex RNA was found in five suspected dengue cases from disease surveillance. The rRT-PCR assays identified VEEV complex RNA in three *Culex* (*Melanoconion*) *vomerifer* pools, leading to VEEV isolates in two. Phylogenetic analysis revealed the VEEV ID subtype in positive samples. Notably, 11.9% of dengue-like disease patients showed VEEV infections. Together, our rRT-PCR validation in human and mosquito samples suggests that this method can be incorporated into mosquito and human encephalitic alphavirus surveillance programs in endemic regions.

## INTRODUCTION

New World alphaviruses (*Togaviridae*, genus *Alphavirus*) are a diverse group of mosquito-borne viruses that can cause severe disease in humans, including the Venezuelan equine encephalitis virus complex (VEEV complex), Madariaga virus (MADV), and Eastern equine encephalitis virus (1, 2). These persist in sylvatic-enzootic cycles throughout the Americas and are transmitted to humans by *Aedes* spp.*, Psorophora* spp., and *Culex* spp. mosquitoes (2, 3).

Serologic and molecular evidence points to widespread VEEV complex infections in tropical Central and South America, indicating potential commonality yet significant underdiagnosis (2). At least 14 different viral subtypes within the VEEV complex have been identified to date (2), some associated with large equine and human outbreaks (VEEV subtypes IAB and IC) (1, 2). While most infections in humans are asymptomatic or subclinical, patients may develop acute febrile illness with headache, myalgias, arthralgias, nausea, and vomiting (4, 5). Cases can progress to encephalitis and result in long-term neurological effects (5, 6).

MADV, once considered a variant of EEEV, is an emerging virus that was first associated with large outbreaks in 2010 in the Darien province of Panama (5), where VEEV subtype ID has also been detected (7). MADV was primarily linked to equine disease, with a few human cases in Trinidad and Tobago and Brazil before the Panama outbreak (8, 9). This contrasts with North American EEEV, associated with severe and fatal human cases (3). MADV detection methods are limited, and its prevalence outside Darien province is not well understood (10). MADV’s geographic expansion to Northeast Brazil and Haiti highlights its potential for new areas (11, 12).

Accurate detection of VEEV complex, MADV, and EEEV during the acute phase is hindered by non-specific clinical signs and limited diagnostic tools. Antigen-based methods are unavailable, and serology requires paired samples to confirm diagnosis (1, 5). Current molecular tests lack optimal performance characteristics necessary for routine testing (13 – 19), and assay design is challenged by VEEV complex genetic variability (2). VEEV complex and MADV are often misdiagnosed as dengue virus due to similar symptoms during the acute phase (2). Common molecular tests involve pan-alphavirus primers amplifying a 400–500 nucleotide genome region, followed by sequencing or nested PCR for identification (5, 13, 14, 16, 18 – 20). These methods are labor-intensive and prone to contamination. Pan-alphavirus primers and conventional reverse transcription polymerase chain reaction (RT-PCR) chemistry may be less sensitive than real-time RT-PCR (rRT-PCR), with few reported rRT-PCR methods differentiating the VEEV complex and MADV (21).

The study aimed to design rRT-PCRs for the VEEV complex and MADV, with a secondary goal of developing a duplex MADV/EEEV rRT-PCR. These assays were evaluated using clinical samples from a Panama alphavirus outbreak and disease surveillance. Additionally, viral species, subtype, and genotype characterization were done using metagenomic sequencing on rRT-PCR-positive samples from humans and mosquitoes collected during the 2015 and 2022 outbreaks in Panama.

## MATERIALS AND METHODS

### VEEV complex, EEEV and MADV rRT-PCR design

Distinct alignments were established for the VEEV complex, EEEV, and MADV using comprehensive genome sequences from the NCBI GenBank (22) and aligned with MegAlign software (DNASTAR, Madison, WI, USA). The VEEV complex alignment encompassed complete genomes from Cabassou, Everglades, Mosso das Pedras, Mucambo, Pixuna, Rio Negro, Tonate, and VEEV subtypes (IAB, IC, ID, and IE). This compilation occurred in 2016 (*n* = 121 sequences), with a similar one for MADV in 2019 (*n* = 32). Employing Primer3 software (primer3.ut.ee), primers and probes were designed to contain ≤1 degenerate base and to align ≥95% with available sequences for each virus (Table 1). *In silico* validation details can be found in supplemental material.

**TABLE 1:** Primers and probes in the VEEV and MADV/EEEV rRT-PCRs^*a*^

Name	Sequence^*b*^	Concentration (nM)^*c*^	Location (5′−3′)^*d*^	Sequences fully matching^*e*^
VEEV				
VEEV forward 1	GAAAGTTCACGTTGAYATCGAGGA	200	44–67	156/159 (98)
VEEV forward 2	GAAGGTTCACGTTGAYATCGAGGA	200		
VEEV reverse 1	GCTCTGGCRTTAGCATGGTC	200	144–163	159/159 (100)
VEEV reverse 2	GCTCTAGCRTTAGCATGGTC	200		
VEEV probe	5′-FAM-TTGAGGTAGAAGCHAAGCAGGTC-BHQ-1–3′	400	112–134	158/159 (99)
MADV/EEEV				
ME forward	GAGATAGAAGCMACGCAGGTC	400	121–141; 99–119	31/32 (97); 1/449 (100)
ME reverse	TGYTTGGAATGCGTGTGC	400	255–272; 233–250	32/32 (100); 9/449 (98)
MADV probe	5′-FAM-CATCGAAAGCGAAGTGGACC-BHQ-1–3′	200	195–214	31/32 (97)
EEEV probe	5′-CFO560-TGAGGGAGAAGTGGAYACAGACC-BHQ-1–3′	400	176–198	6/449 (99)

^*a*^ BHQ, black hole quencher; CFO560, CAL Fluor Orange 560; and FAM, Fluorescein.

^*b*^ Probe sequences listed 5’′-fluorophore-sequence-quencher-3’′.

^*c*^ Concentration in the final reaction mixture.

^*d*^ Location in the following complete genome sequences: VEEV strain VEEV/Homo sapiens/GTM/69Z1/1969/IAB (Aaccession number KC344505.2); MADV strain Homo sapiens/Haiti-1901/2016 (MH359233.1); EEEV strain EEEV/Culiseta melanura/USA/SL13-0764-C/2013 (Aaccession number KX029319.1).

^*e*^ Displayed as number of complete genome sequences without a mismatch in the primer/probe sequence over all complete genome sequences aligned (%). Genomes downloaded on 22 Sept. 2021. Data shown for the combination of forward and reverse VEEV primers.

### rRT-PCR assay performance and optimization

**Fig 1:**
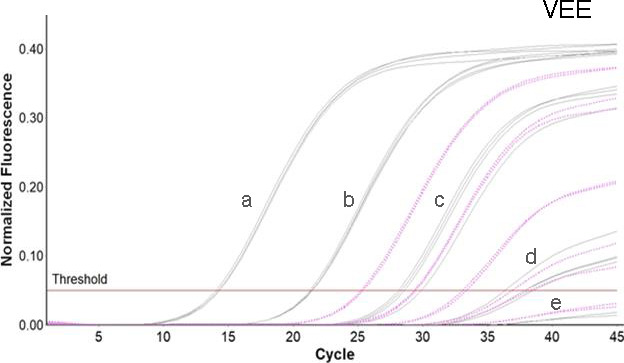
VEE amplification curves across a range of concentrations. Amplification curves are shown across a range of concentrations for the VEE complex rRT-PCR with ssDNA (gray curves, subtype IAB) and RNA (pink dotted curves, subtype IC). ssDNA was tested in quadruplicate at 8.0, 6.0, 4.0, 2.0, and 1.0 log_10_ copies/µL (labeled a–e, respectively). 10-fold dilutions of VEEV subtype IC RNA were tested in duplicate starting at the highest concentration available (5.0 log_10_ copies/µL).

Primer and probe sets were evaluated in singleplex reactions containing 200 nM of each oligonucleotide and genomic RNA or quantified ssDNA containing the target region. Primer/probe sets were selected to generate the most sensitive detection based on cycle threshold (Ct) values, with preserved specificity. Primer and probe concentrations in the final reaction were then adjusted between 100 and 400 nM to optimize assay sensitivity. For VEEV, a total of four primers are mixed in a single reaction (Table 1). Additional validation, conditions, and lower limit of detection (LLOD) are given in the supplemental material (Fig. 1).

### Protocol validation with acute human samples

**Fig 2:**
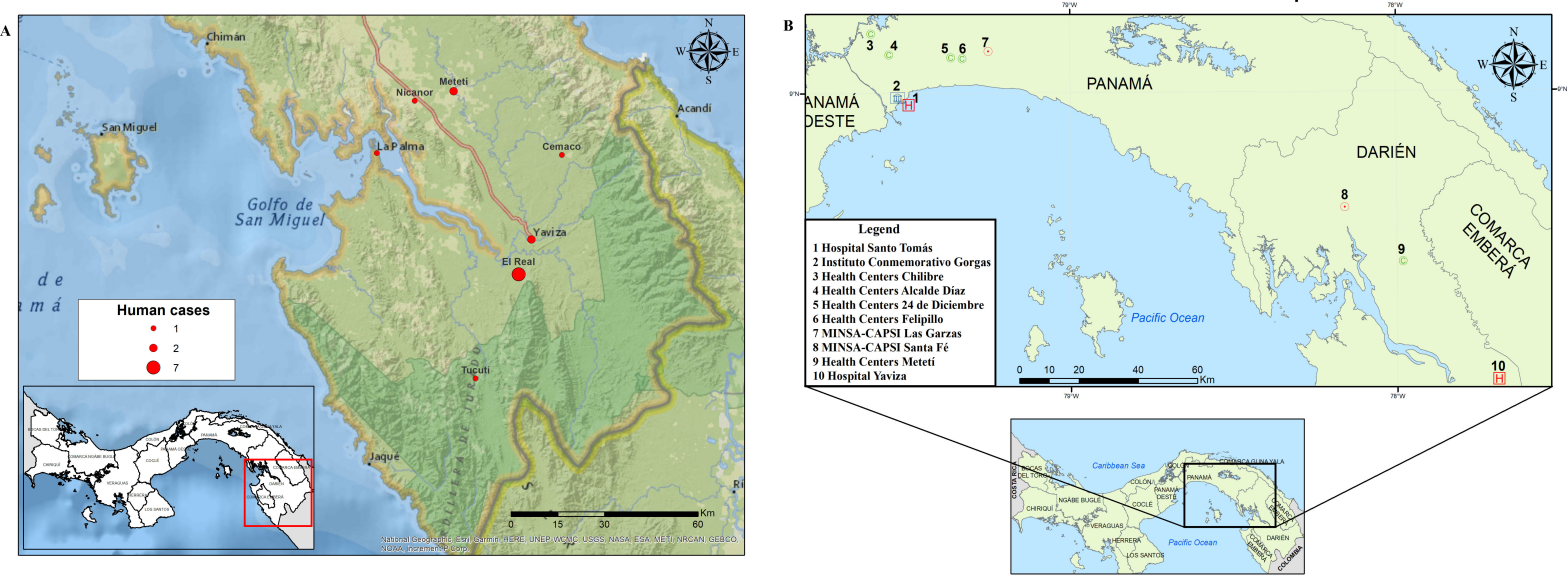
Map with the distribution of VEEV human cases in Darien province in 2015 and 2017, and health centers in Panama and Darien provinces. (A) Distribution of VEEV cases used for protocol validation. Red dots represent the number of cases reported by locality. (B) Distribution of health centers used for prospective febrile surveillance in Panama and Darien provinces. The map was created with ArcGIS Desktop 10.6 using shapefiles from Esri [World Countries Generalized (2021); https://www.arcgis.com/home/item.html?id=2b93b06dc0dc4e809d3c8db5cb96ba69]. Data sources for the shapefiles include Esri, Garmin International Inc., U.S. Central Intelligence Agency, and National Geographic Society (24).

Acute human samples used in the protocol validation were collected in communities of Darien, the easternmost province in Panama, during three alphavirus outbreaks in 2015 and 2017. Cases identified in 2015 and 2017 were detected in the communities of Metetí, Cemaco, Tucutí, Yaviza, Nicanor, La Palma, and El Real de Santa María (Fig. 2A). The Darien province borders Colombia and encompasses the Darien Gap, and the Darien National Park, a UNESCO-designated World Heritage Site (23).

### Patient recruitment in 2015 and 2017

Febrile patients were identified during an enhanced surveillance program by our outbreak response team using house-by-house visits during the 2015 and 2017 outbreaks. Blood samples were drawn from patients who met the case definition during the outbreak investigation.

### Prospective acute disease surveillance in 2021 and 2022

In 2021, surveillance for emerging pathogens was established in Panama as part of the USA-National Institute of Allergy and Infectious Diseases, Centers for Research in Emerging Infectious Diseases Network initiative. The Coordinating Research on Emerging Arboviral Threats Encompassing the Neotropics (CREATE-NEO) in Panama undertakes acute febrile surveillance across 10 health centers in Panama and Darien Provinces (Fig. 2B) (https://www.utmb.edu/createneo/home/create-neo-home). Additional information of inclusion criteria is provided in the supplemental material.

### Laboratory testing for acute disease surveillance

Acute samples (0–5 days) were first screened against DENV, CHIKV, and ZIKV virus using rRT-PCR as described previously (25), followed by testing with the newly designed MADV/VEEV rRT-PCR.

### Mosquito collection

Mosquitoes were collected in a forested area (100 × 100 meters) in El Real de Santa María during the 2015 outbreak response. CDC light traps were employed over a 12-hour period (6:00 p.m. to 6:00 a.m.), positioned 1.5 meters above the ground level. These traps, equipped with octanol and CO2 as bait, were utilized for the encephalitis vector survey. Captured mosquitoes were anesthetized, identified to species using taxonomic keys (26), and preserved in liquid nitrogen. Specimens were grouped by species, with a maximum of 20 individuals per pool for subsequent analyses.

### Viral isolation from mosquito pools

Mosquito pool homogenates were prepared with 20–50 mosquitoes in 2 mL of minimum essential medium supplemented with penicillin and streptomycin, and 20% fetal bovine serum (FBS), homogenized using a Tissue Lyser (Qiagen, Hidden, Germany) and centrifuged at 12,000 rpm for 10 min. A total of 200 µL of serum or mosquito homogenate was inoculated in each of two 12.5-cm^2^ flasks of Vero cells (*Cercopithecus aethiops* kidney normal cells, ATCC CCL-81). Vero cells were supplemented with 10% FBS for growth and maintained with 2% FBS and 1% penicillin/streptomycin at 37°C. Samples were passed twice and monitored for cytopathic effect. All viral isolations were undertaken in the biosafety level-3 containment laboratory at the Gorgas Memorial Institute in Panama City.

### Generic alphavirus RT-PCR for human and mosquito samples

Viral RNA was extracted from human serum and mosquito pool homogenates using QIAamp RNA viral extraction kit (Qiagen, Valencia, CA, USA). Viral RNA from mosquitoes was also extracted using the Macherey-Nagel extraction kit (Düren, Germany). Volume for extraction was 160 and 200 µL for human serum and mosquito pool homogenates, whereas elution volume was 60 and 50 µL, respectively. Serum and mosquito homogenates were tested in 25 µL reactions for alphaviruses using a universal alphavirus RT-PCR, as previously described (19). Antibody response was assessed in all human serum samples from 2015 as described previously (27), further details are provided in the supplemental material.

### Viral metagenomic sequencing

To confirm virus species, subtype, and genotype, we sequenced seven selected VEEV complex rRT-PCR positive mosquito and human samples from 2015 and 2022 using SMART-9N metagenomic sequencing as previously described (28). Additional information is provided in the supplemental material.

### VEEV phylogenetic analysis

All available VEEV genome sequences, in GenBank, representing all antigenic complexes were selected to construct the alignment; duplicated sequences, partial sequences, and overlapping sequences were removed. Finally, the novel complete or near complete VEEV genome sequences (*n* = 7) were aligned with 132 representative VEEV genomes retrieved from NCBI GenBank using MAFFT version 7 (29). Selection of the best-fitting nucleotide substitution model and maximum likelihood phylogenetic reconstruction were performed with IQ-Tree v2.2.0.3 (30). Statistical robustness of the tree topology was assessed with 1,000 ultrafast bootstrap replicates.

## RESULTS

### rRT-PCR analytical evaluation

Primers and probes for the VEE complex singleplex and MADV/EEEV duplex rRT-PCRs are shown in Table 1 along with the optimized final reaction concentrations. The dynamic range for each assay extended from 2.0 to 8.0 log_10_ copies/µL (Fig. 1; Fig. S1). For the VEEV complex assay, the linear range was evaluated with ssDNA for subtypes IAB and IV and RNA from subtype IC (2.0–5.0 log_10_ copies/µL; Figure 2). The 95% LLODs, expressed in copies/µL, were: VEEV subtype IAB, 120; VEE subtype IV, 110; MADV, 19; and EEEV, 19. Assay exclusivity was evaluated by testing genomic RNA from VEEV subtype IC, EEEV, and a set of arboviruses, including flavi-, bunya-, and alphaviruses on a single run of the VEEV complex and MADV/EEEV rRT-PCRs. VEEV complex and EEEV only yielded signals in the respective assays for these viruses. None of the other tested viruses generated a signal in either assay. In addition, none of the 56 serum samples from Georgia, USA, or Asunción, Paraguay, tested positive in either assay.

### Validation with clinical samples

A total of 15 febrile patients from the 2015 and 2017 alphavirus outbreaks who met the suspected or probable case definition were used to validate the new molecular assays. Previously, a total of eleven (11/15) acute serum samples collected during the 2015 and 2017 alphavirus outbreaks had tested positive using a generic alphavirus RT-PCR and were confirmed later by sequencing as VEEV-ID infections (17). In 2021, a second round of generic alphavirus RT-PCR using the same set of primers was run on these 15 stored samples, and all of them tested negative. Notably, using the newly designed rRT-PCR, we were able to detect 10 VEEV complex RNA positive samples (Ct range: 27–38), including two samples that had tested negative at the initial screening in 2017 (Table 2). Three of the VEEV complex rRT-PCR-positive samples were also anti-VEEV IgG and IgM positive, with only 0, 2, and 3 days since the onset of symptoms, respectively (Table 2). One sample was rRT-PCR positive for both VEEV and MADV viruses.

**TABLE 2:** Characteristics and laboratory results of the samples used for protocol validation patients and clinical samples and laboratory results^*a*^

Code	Township	Age^*b*^	Sex	Symptoms onset	Days of symptoms	RT-PCR-alpha (2015)	RT-PCR-alpha (2021)	rRT-PCR-VEE	Ct values	IgM-VEEV	IgM-MADV	IgG-VEEV	IgG-MADV	PRNT-veev^*c*^	PRNT-MADV^*c*^
258384	El Real	0–9	M	Aug. 2015	0	**pos**	neg	**pos**	29.3	neg	neg	neg	neg	<1:20	<1:20
267738	Cemaco	0–9	M	July 2017	3	neg	neg	**pos**	37.8	**pos**	neg	neg	neg	<1:20	<1:20
267411	Tucuti	0–9	F	July 2017	5	neg	neg	neg	–^*d*^	pos	neg	pos	pos	1:40	1:40
258380	El Real	0–9	F	Aug. 2015	1	**pos**	neg	neg	–	neg	neg	neg	neg	<1:20	<1:20
267410	Yaviza	0–9	F	July 2017	2	neg	neg	neg	–	pos	neg	pos	neg	<1:20	<1:20
258657	Yaviza	10–19	M	Sept. 2015	0	**pos**	neg	**pos**	28	neg	neg	neg	neg	<1:20	<1:20
258535	Nicanor	20–29	F	Sept. 2015	2	**pos**	neg	neg	–	neg	neg	neg	neg	<1:20	<1:20
258401	La Palma	20–29	M	Aug. 2015	2	**pos**	neg	**pos**	29	neg	neg	neg	neg	<1:20	<1:20
258395	Metetí	30–39	M	Aug. 2015	2	neg	neg	**pos**	37	**pos**	neg	neg	neg	<1:20	<1:20
258399	El Real	30–39	M	Aug. 2015	1	**pos**	neg	**pos**	26	neg	neg	neg	neg	<1:20	<1:20
258385	El Real	30–39	M	Aug. 2015	2	**pos**	neg	**pos**	37	neg	neg	neg	neg	<1:20	<1:20
258398	El Real	30–39	M	Aug. 2015	0	**pos**	neg	**pos**	27	neg	neg	**pos**	neg	<1:20	<1:20
258536	Metetí	30–39	F	Sept. 2015	2	**pos**	neg	neg	–	neg	neg	neg	neg	<1:20	<1:20
258386	El Real	30–39	M	Aug. 2015	5	**pos**	neg	**pos**	34	neg	neg	neg	neg	ND	ND
258379	El Real	≥40	F	Aug. 2015	2	**pos**	neg	**pos**	31	neg	neg	neg	neg	<1:20	<1:20

^*a*^ Acute samples selected from the 2015 and 2017 alphavirus outbreaks in Darien Province. Ct, cycle threshold; neg, negative; pos, positive; and ND, not done.

^*b*^ Age categories in years.

^*c*^ Base on PRNT-80.

^*d*^ -, not done and postive results and higligthed in bold.

### Prospective disease surveillance

**Fig 3:**
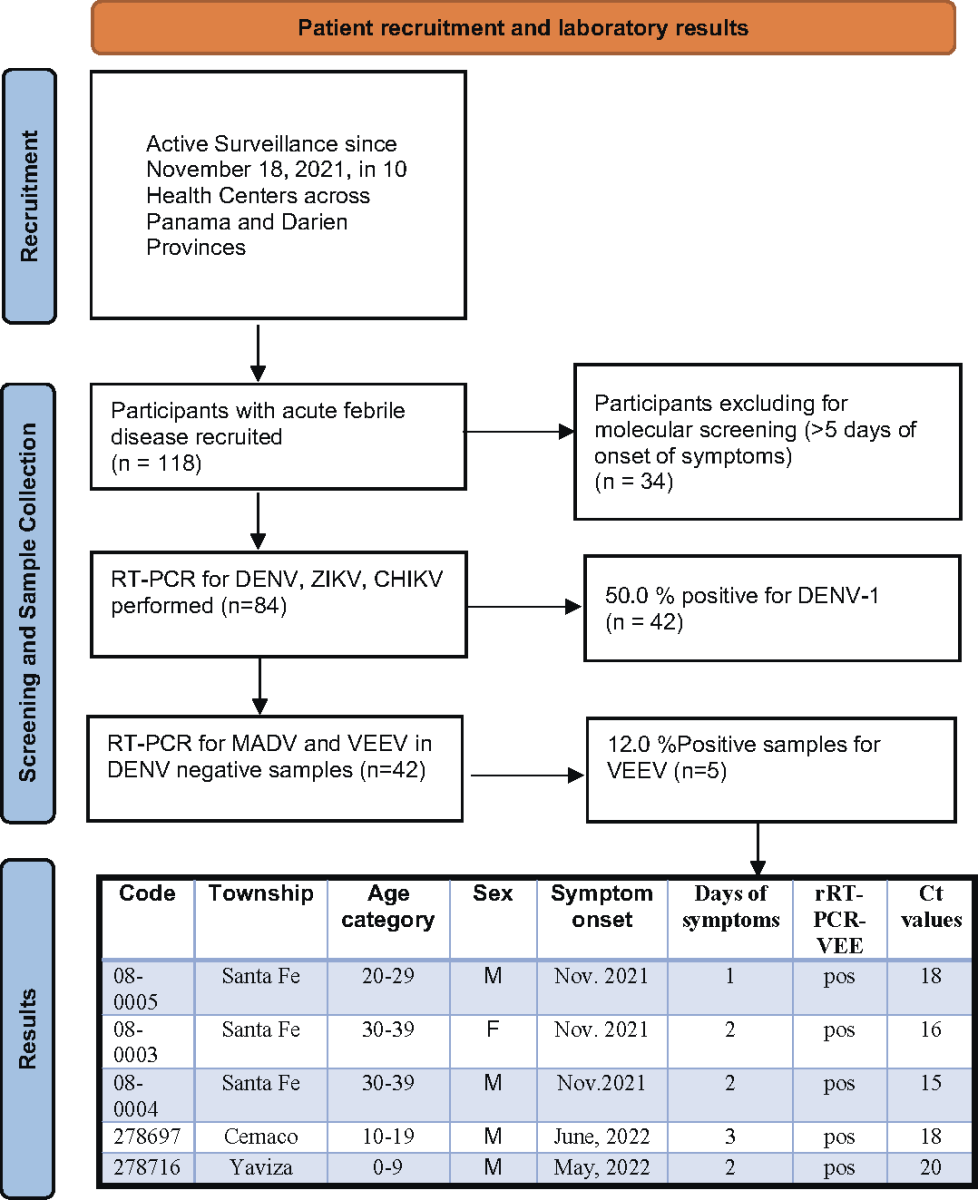
Flowchart of patient recruitment, characteristics, and RT-PCR results of febrile patients detected throughout disease surveillance. Febrile patients were recruited from 16 November 2021 to 1 December 2022, in 10 health care centers in Panama and Darien provinces.

A total of 118 febrile patients were recruited from 16 November 2021 to 1 December 2022. Of these, 84 (71.2%) were acute patients with the onset of symptoms ranging from 0 to 5 days. A total of 42 patients (50.0%) were DENV1 positive. We detected VEEV RNA (Ct range: 15–20) in five patients (11.9%; 95% CI: 4.0–25.6) with suspected dengue infection, one of which was from a fatal case in 2022. Details and results of disease surveillance are presented in Fig. 3.

### Viral detection in mosquito pools

A total of 1,307 mosquitoes belonging to 35 species and 12 genera were collected in the community of El Real de Santa Maria, Panama, during a period of 5 days in 2015 (Table 3). The most abundant mosquito species was *Coquilletidia venezualensis* (37.5%, *n* = 490 of 1,307) and *Culex Melanoconion vomerifer* (34.4%, *n* = 450 of 1,307). Mosquito species, number of individuals, and pools are shown in Table 3. Of 150 mosquito pools, 3 *Cx*. (*Mel.) vomerifer* mosquito pools tested positive for VEEV by rRT-PCR (Ct range: 26–30). Two of these rRT-PCR-positive pools also yielded viral isolates.

**TABLE 3:** Mosquito species collected during the 2015 outbreak in El Real de Santa Maria, Panama

Mosquito species	N	(%)	Pools^*a*^	VEE-rRT-PCR positive	MADV-rRT-PCR positive	Viral isolates
*Coquillettidia venezuelensis*	490	37.5	29	0	0	0
*Culex (Melanoconion) vomerifer*	450	34.4	27	3	0	2
*Culex (Melanoconion) pedroi*	32	2.4	4	0	0	0
*Aedes serratus*	31	2.4	7	0	0	0
*Aedes* sp.	30	2.3	5	0	0	0
*Culex (Melanoconion*) sp.	30	2.3	6	0	0	0
*Culex (Culex) interrogator*	27	2.1	5	0	0	0
*Anopheles trianulatus*	23	1.8	2	0	0	0
*Aedes eupoclamus*	14	1.1	4	0	0	0
*Culex (Culex) nigripalpus*	14	1.1	3	0	0	0
*Culex (Culex*) sp.	14	1.0	4	0	0	0
*Culex (Melanoconion) atratus*	14	1.0	1	0	0	0
*Culex (Melanoconion) adamesi*	13	1.0	3	0	0	0
Others^*b*^	125	9.6	50	0	0	0
Total	1307	100	150	3	0	2

^*a*^ Numbers of mosquito pools.

^*b*^ Species <1% abundance are listed as others.

### VEEV subtype identification

**Fig 4:**
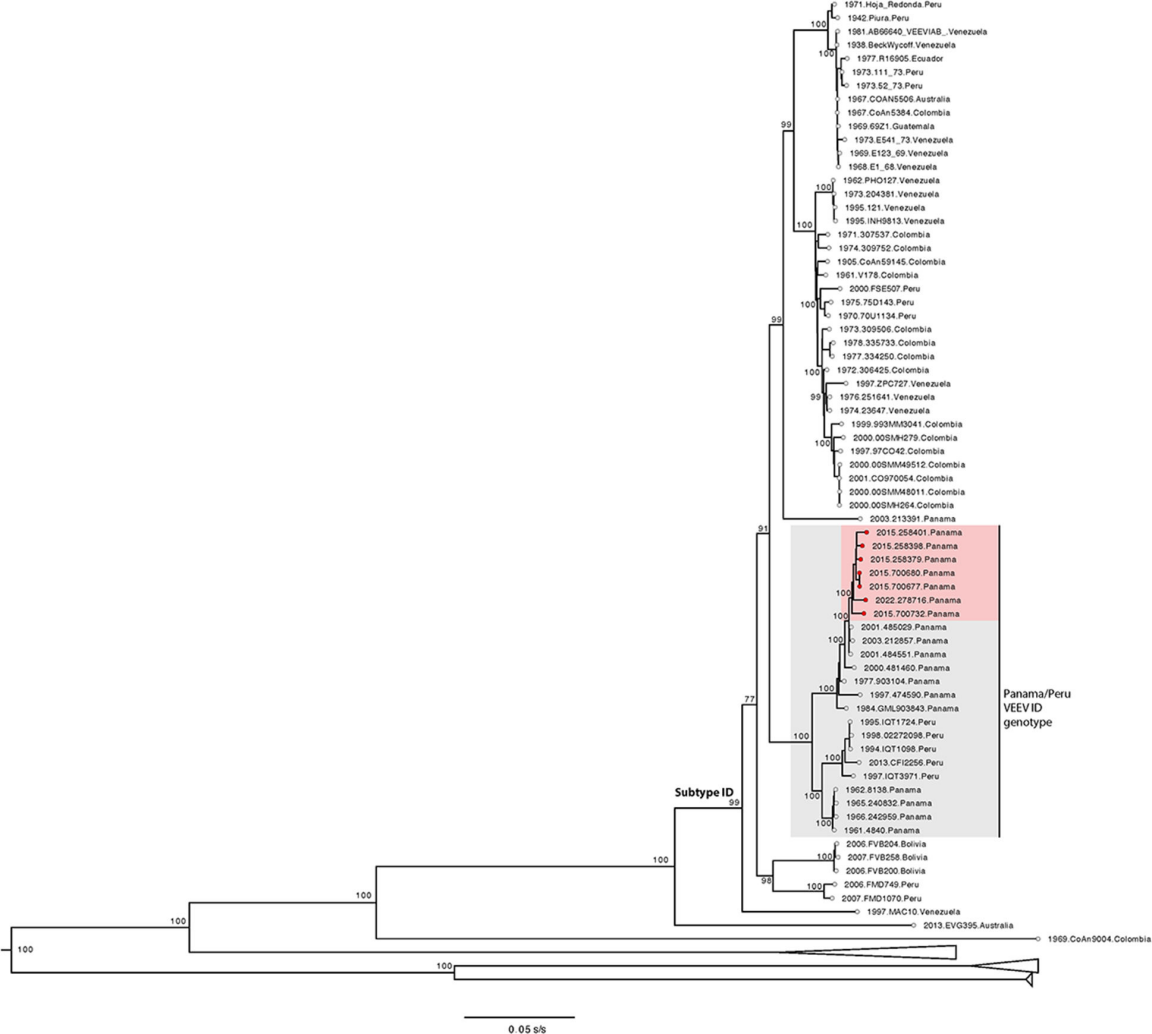
VEEV complex maximum likelihood phylogenetic tree. Maximum likelihood phylogeny was estimated using 139 complete or near complete VEEV genomes. Publicly available Panamanian VEEV ID subtype strains are highlighted in gray (*n* = 15), and genomes generated in this study (*n* = 7) are highlighted in red. Bootstrap statistical support is shown for selected nodes. NCBI GenBank accession numbers for the new VEEV genomes are: OR644785, OR644786, OR644788, OR644801, OR644802, OR644803.

Three mosquito pools and four human samples (including one from a fatal case in 2022), which tested positive for the new VEEV complex rRT-PCR, were sequenced using a virus-untargeted approach (28). The 20-fold genome coverage per base pair ranged from 45% to 100% (Table 4). The percentage of genome identity with the VEEV reference strain ranged from 87.7% to 90.0% (Table 4), while identity with the Panamanian VEEV ID subtype prototype strain 3880 ranged from 96% to 97% (Table 4). Maximum likelihood phylogenetic analysis indicated that the new viral genomes cluster together with historical Panamanian VEEV ID subtype strains within the Panama/Peru genotype (bootstrap statistical support = 100; Fig. 4).

**TABLE 4:** Metadata and sequencing statistics for selected VEEV complex RNA positive samples^*c*^

ID	Collection year	Location	Host species	Percent genome coverage 20×	Percent Nt identity with genome reference^*a*^ ^,*b*^	Percent identity with strain 3880^*c*^	*C* values
**700677**	2015	Darien	*Culex* (*Mel.*) *vomerifer*	100	89.8	92.1	27
**700680**	2015	Darien	*Culex* (*Mel.*) *vomerifer*	100	89.8	92.2	31
**700732**	2015	Darien	*Culex* (*Mel.*) *vomerifer*	100	90	92.3	26
**258379**	2015	Darien	Human	99.9	89.6	92	31
**258398**	2015	Darien	Human	70	88.7	90.7	27
**258401**	2015	Darien	Human	90.6	87.7	90	29
**278716**	2022	Darien	Human	45.98	88.1	90	20

^*a*^ Genbank accession no. NC_001449.1.

^*b*^ Nt = Nucleotide.

^*c*^ Panamanian VEEV ID subtype prototype strain 3880, GenBank accession no. L00930.1.

## DISCUSSION

Encephalitic alphaviruses have been detected throughout the Americas and may account for a significant proportion of non-dengue acute febrile illness (1, 2, 5, 9). Assays for their molecular detection, although existing (14 – 20), are often time-consuming, involving multiple PCR rounds or subsequent genome sequencing limited to well-equipped facilities (14 – 20). Co-circulation and the potential for co-infection with these viruses further complicate their identification, especially when clinical presentations are similar, and convenient methods for detecting VEEV complex and MADV are lacking (19). In Panama, for instance, both VEEV subtype ID and MADV have been identified, with co-circulation detected along the Colombian border (5 – 7). Typically, cases are identified during the neurological phase of the disease (5, 31), where the virus is cleared from serum, necessitating reliance on serological testing. Given that alphaviruses can induce IgM responses lasting 2–3 months, anti-VEEV or anti-MADV IgM detection alone could lead to misdiagnosis without seroconversion (5, 31).

We have developed singleplex and duplex rRT-PCRs for detecting VEEV complex, MADV, and EEEV viral RNA in clinical and mosquito samples. These assays identified VEEV ID subtype and MADV in samples previously negative using a reference RT-PCR (19). We also identified a VEEV ID subtype—MADV co-infection, highlighting an advantage of our VEEV complex and MADV/EEEV rRT-PCRs over prior methods. Co-infection cases are epidemiologically significant and may have clinical relevance if associated with more severe disease (5). Our rRT-PCR assays can be rapidly integrated into testing algorithms in endemic regions. The current rRT-PCR detects VEEV ID subtype RNA within the initial 5 days of symptoms, preceding IgM and IgG antibody responses which usually manifest after 5–7 days following symptom onset (32). Intriguingly, three patients with detectable VEEV complex RNA were also VEEV IgM and IgG-reactive, suggesting possible VEEV re-infections with potential implications for vaccine development. However, early IgM responses cannot be ruled out, necessitating further research on alphavirus humoral immunity.

Through our prospective disease surveillance in Panama, we have demonstrated a notable prevalence of alphavirus detection. About 11.9% of individuals exhibiting symptoms similar to dengue have been found to have VEEV infections. These findings align with earlier assessments indicating that roughly 10% of clinical dengue cases in endemic countries can be attributed to VEEV infection (2). Moreover, this suggests a co-circulation of alphaviruses alongside other endemic arboviral infections, including dengue. Given the clinical similarities between VEEV complex infections and dengue, there exists the potential for underestimating the true burden of VEEV-related disease (2).

VEEV ID subtype RNA was found in *Cx*. (*Mel*.) *vomerifer* mosquito pools trapped during the 2015 outbreak in El Real de Santamaria. These mosquitoes were previously implicated as VEEV ID subtype vectors (2). Two pools yielded viral isolates. Notably, pan-alphavirus conventional RT-PCRs failed to detect viral RNA in these pools, suggesting the new rRT-PCR’s heightened sensitivity for VEEV complex RNA detection in mosquitoes. Neither MADV nor EEEV infections were detected in mosquitoes using various methods. A similar pattern emerged from past outbreak investigations by our group in Panama (27, 33). Interestingly, MADV detection frequency in *Culex* (*Mel*.) spp. mosquitos is low in Panama (34, 35), unlike the endemic region of Iquitos, Peru, where MADV in the enzootic vector *Culex* (Mel.) *pedroi* is frequent (9, 36). Reasons for this variation in MADV and VEEV ID subtype frequency in Panama and MADV and VEEV in Panama vs Iquitos remain uncertain, possibly involving vector competence or viral competition, even enhanced VEEV ID subtype transmission via insect-specific viruses (37).

While our assays were validated with a limited number of human and mosquito samples, prospective surveillance allowed further validation. Unlike previous methods relying on plasmids, viral isolates, or a few human serum samples (13 – 21), we validated with human serum, mosquitoes, and post-mortem tissue samples. Our approach failed to detect two samples previously positive using standard alphavirus generic primers (19). Interestingly, a subsequent generic alphavirus RT-PCR in 2017 also failed to reamplify the former positives, possibly due to viral RNA degradation over time (38)

An rRT-PCR based on 33 VEEV sequences was reported by Vina-Rodriguez et al. but excluded other VEEV complex species and lacked clinical evaluation (21). Our assays used more complete genome sequences, with *in silico* primer and probe alignment to contemporary sequences. Untargeted metagenomic sequencing confirmed VEEV ID subtype detection using VEEV complex primers; this subtype has been detected in central and eastern Panama regions (7). These findings highlight molecular and genomic approaches’ potential to enhance the detection of acute encephalitis alphavirus infections, even in archived samples.

Further prospective testing is necessary for comprehensive clinical performance characterization, including quantitative diagnostics and challenging assays with interfering substances. Limitations include the design requiring two separate assays for three viruses due to overlapping optimal design targets. However, the two rRT-PCRs can be executed simultaneously, improving lab workflow. The VEEV complex assay can also be multiplexed with rRT-PCRs for other neurotropic arboviruses without performance loss (Jesse J. Waggone, unpublished data).

We developed sensitive and specific VEEV complex, MADV, and EEEV rRT-PCRs, surpassing available molecular methods. These assays detect VEEV-MADV co-infections, VEEV human infections, potential VEEV reinfections, and active VEEV viral circulation in mosquitoes during alphavirus outbreaks. Implementing these assays in endemic regions may enhance neurotropic alphavirus identification and characterization.

## Data Availability

All the data used for human and mosquito validation are contained within the manuscript. Accession numbers for the newly generated genomes are OR644785, OR644786, OR644788, OR644801, OR644802, OR644803. Accession numbers and strain information of sequences used for primer design are shown in the supplemental material.

## SUPPLEMENTAL MATERIAL

Supplemental file 1. Additional information. jcm.00152-23-s0001.docx Addicional information of recruitment and methods used in the study.

Table S1. Accession Numbers jcm.00152-23-s0002.xlsx Genome accession numbers used in primer design.

ASM does not own the copyrights to Supplemental Material that may be linked to, or accessed through, an article. The authors have granted ASM a non-exclusive, world-wide license to publish the Supplemental Material files. Please contact the corresponding author directly for reuse.
