# Supplementary material for: Real-time RT-PCR for Venezuelan equine encephalitis complex, Madariaga, and Eastern equine encephalitis viruses: application in human and mosquito public health surveillance in Panama
Source: J Clin Microbiol. 2023 Nov 20;61(12):e00152-23. doi: 10.1128/jcm.00152-23 (PMC10729654; doi:10.1128/jcm.00152-23)
Supplement: Supplemental file 1. Additional information. — Addicional information of recruitment and methods used in the study. [file jcm.00152-23-s0001.docx]

Suppementary materials for:

**Real-time RT-PCR for Venezuelan equine encephalitis complex, Madariaga and Eastern equine encephalitis viruses: application in human and mosquito public health surveillance**

**rRT-PCR in silico validation**

In silico primer/probe specificity was checked by aligning sequences in BLAST (blast.ncbi.nlm.nih.gov) against (i) all available sequences and (ii) only alphavirus sequences while excluding the VEEV complex or MADV, respectively. Due to the similarity between MADV primers and EEEV sequences, all available EEEV complete genome sequences (n=441) were aligned and separate MADV and EEEV probes were designed for an rRT-PCR duplex assay. Alignments for each virus were repeated with all sequences available in September 2021 to confirm primer and probe sequences in contemporary strains.

**rRT-PCR optimization**

Primers were obtained from Integrated DNA Technologies (IDT, Coralville, Iowa); probes were obtained from Biosearch Technologies (Hoddesdon, United Kingdom). VEEV subtype IC and EEEV genomic RNAs were purchased from Vircell Microbiologists (Granada, Spain). Quantified Ultramer ssDNA containing the assay target region was obtained for all viruses (IDT, Coralville, Iowa) to evaluate assay performance. For ssDNA synthesis, target region sequences were selected from specific strains of VEE subtype IAB (Accession number KC344505.2) and subtype IV (Pixuna virus, Accession number NC_038673.1), MADV (Accession numbers MH359233.1 and KJ469626.1), and EEEV (Accession number KX029319.1).

**rRT-PCR reaction and cycling conditions**

rRT-PCRs were performed in 25 µL reactions using the SuperScript III Platinum One-Step Quantitative RT-PCR Kit (Thermo Fisher, Waltham, MA) with 5 µL of the nucleic acid template. The analytical evaluation was performed on a Rotor-Gene Q instrument (Qiagen, Germantown, MD), and the validation with serum and mosquito pool samples was performed on an ABI7500 (Thermo Fisher). Cycling conditions were consistent with previous laboratory protocols: 52 °C × 15 min, 94 °C × 2 min, and 45 cycles of 94 °C × 15 s, 55 °C × 40 s (acquired in all channels), and 68 °C × 20 s (1–3). rRT-PCR thresholds were set based on testing with the final reaction mixtures, as described previously (1, 3). The dynamic range of each assay was determined by testing synthesized targets from each reference strain in quadruplicate at 8.0, 6.0, 4.0, 2.0, and 1.0 log_10_ copies/μL. For the VEEV complex, the lower limit 95% detection (95% LLOD) was estimated by testing 10 replicates of 2-fold serial dilutions from 200 to 25 copies/μl. For MADV and EEEV, 95% LLOD was estimated from results of replicate testing in the dynamic range study. Probit analyses were performed using MedCalc, v20.013 (MedCalc Software, Belgium) to estimate LLOD as previously described(1, 3).

**rRT-PCR Assay Specificity**

Specificity was evaluated by testing 56 serum samples from locations without known transmission of VEEV or MADV. These included 8 samples collected from patients in Georgia, USA, without known travel history, and 48 samples from individuals with an acute febrile illness in Asunción, Paraguay. The latter samples have been described in detail elsewhere (2). Total nucleic acids were extracted from 200µL of serum on an EMAG instrument (BioMérieux, Durham, NC), eluted in 50 µL and tested with the VEEV complex and MADV/EEEV rRT-PCRs.

**rRT-PCR Assay exclusivity**

Assay exclusivity was evaluated by testing genomic RNA from the following viruses (strain in parentheses, if designated): Rift Valley fever (h85/09); Zika (ZIJV; MR766); dengue virus serotype 1 (DENV1, Hawwai 1944), DENV2 (NGC), DENV3 (Sleman/78), and DENV4 (H241); chikungunya virus (CHIKVR80422); Mayaro virus (MAYV; ARV 0565, INHRR 11a-10); yellow fever virus (YFV; 17D and Asibi strains); West Nile virus (WNV; NAL); St. Louis encephalitis virus (SLEV; GML 902612, CorAn 9275); tick-borne encephalitis virus (TBEV; Japanese encephalitis virus (JEV); Semliki Forest virus (SFV); Ross River virus (RRV); Getah virus (GETV); Barmah Forest virus (BFV); and Una virus (UNAV).

**Outbreak case definition**

The definition of a suspected case included fever and headache, while a probable case was defined as a suspected case plus somnolence, lethargy, or convulsions. Blood samples were centrifuged in the field, and serum was stored in liquid nitrogen for transportation to the Gorgas Memorial Institute of Health Studies in Panama City.

**Inclusion criteria for Prospective surveillance**

Cases, without malaria, human immunodeficiency virus (HIV), hepatitis B virus (HBV) and hepatitis virus (HCV), and >5 and <75 years old, presenting with no more than 7 days with rash, and at least one of the following symptoms: fever, myalgia, arthralgia, periarticular edema, and conjunctivitis were recruited, evaluated and interviewed, to obtain clinical, and demographics characteristics and ethic consent at each health center.

**Alphavirus serology of 2015 clinical samples**

All human serum samples were tested in duplicate for IgM antibodies to MADV and VEEV antigen using an enzyme-linked immunosorbent assay (ELISA) and confirmed by a plaque-reduction neutralization test (PRNT). For the ELISA, sucrose-acetone antigens were prepared from MADV- (prepared by Dr. Robert Shope at the Yale Arbovirus Research Unit in August 1989) and VEEV- (strain TC-83) infected mouse brain. For the PRNT, we used chimeric Sindbis virus SINV/MADV (derived from Brazilian MADV strain BeAn436087 and shown to be an accurate surrogate for MADV in these assays (4) and TC83, an attenuated vaccine strain of VEEV closely related to subtype ID strains that circulate in Panama (5). The neutralizing antibody titer was determined as the reciprocal of the highest dilution that reduced plaque count by 80% (PRNT_80_).

**Metagenomic sequencing**

Viral RNA was treated to remove residual DNA with TURBO DNase (Thermo Fisher Scientific, USA) and concentrated with Zymo RNA clean & concentrator-5 (Zymo Research, USA) following the protocol instructions. cDNA synthesis and PCR was performed as described previously (6). PCR products were then purified using AMPure XP beads (Beckman Coulter, UK) and quantified according to manufacturer's instructions with Qubit dsDNA High Sensitivity assay (Life Technologies, USA) and Qubit 3.0 instrument (Life Technologies, USA).

cDNAs were pooled using the EXP-NBD104 (1–12) and EXP-NBD114 (13–24) Native Barcoding Kits (ONT, UK). Sequencing libraries were generated using the SQK-LSK109 Kit (ONT, UK). 50 ng of the final libraries were loaded onto FLO-MIN106 flow cells on the MinION device (ONT, UK) and sequenced using MinKNOW with the standard 48-hour run script. FASTQ files were demultiplexed and trimmed using Gruppy V5.0.16. (Oxford Nanopore, Oxford, United Kingdom), and then aligned and mapped to the reference genome (GenBank accession no. NC_001449.1) using minimap2 version 2.28.0 (7)and converted to a sorted BAM file using SaMtools 3 (http://www.htslib.org). NanoStat version1.1.24 (<https://pypi.org/project/NanoStat/>) was used to compute the number of raw reads and minimum contig length to cover 50 percent of the genome (N50) of the aligned reads. Genome visualization was undertaken with Tablet 1.19.05.28 (8), and to compute the number of mapped reads, percentage of genome coverage, and coverage depth. Variants were detected with medaka_variants and the consensus sequence were built using margin_medaka_consensus (Oxford Nanopore, Oxford), United Kingdom. Genome regions with <20x coverage were masked.

**
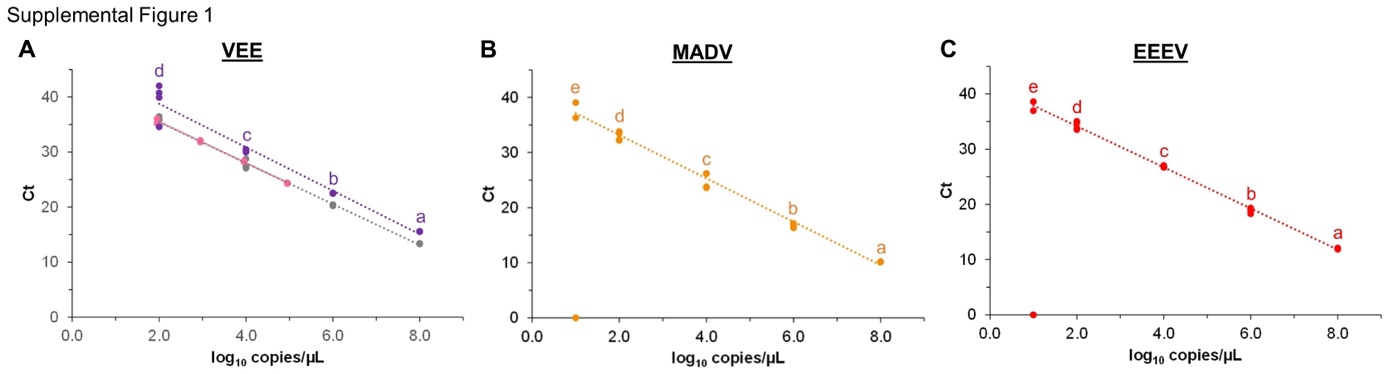
**

**Figure S1. Dynamic range of assays for VEE complex (A), MADV (B) and EEEV (C).** The dynamic range for each assay was established by testing ssDNA in quadruplicate at 8.0, 6.0, 4.0, 2.0 and 1.0 log_10_ copies/µL (labelled a-e, respectively).

**References**

1. Waggoner JJ, Gresh L, Mohamed-Hadley A, Ballesteros G, Vargas Davila MJ, Tellez Y, Sahoo MK, Balmaseda A, Harris E, Pinsky BA. 2016. Single-reaction multiplex reverse transcription PCR for detection of zika, Chikungunya, and dengue viruses. Emerg Infect Dis;22(7):1295-7. doi: 10.3201/eid2207.160326..

2. Rojas A, Cardozo F, Cantero C, Stittleburg V, López S, Bernal C, Gimenez Acosta FE, Mendoza L, Pinsky BA, De Guillén IA, Páez M, Waggoner J. 2019. Characterization of dengue cases among patients with an acute illness, Central Department, Paraguay. PeerJ. 7:e7852. doi: 10.7717/peerj.7852. .

3. Waggoner JJ, Abeynayake J, Sahoo MK, Gresh L, Tellez Y, Gonzalez K, Ballesteros G, Pierro AM, Gaibani P, Guo FP, Sambri V, Balmaseda A, Karunaratne K, Harris E, Pinsky BA. 2013. Single-Reaction, Multiplex, Real-Time RT-PCR for the Detection, Quantitation, and Serotyping of Dengue Viruses. PLoS Negl Trop Dis;7(4):e2116. doi: 10.1371/journal.pntd.0002116.

4. Johnson BW, Kosoy O, Wang E, Delorey M, Russell B, Bowen RA, Weaver SC. 2011. Use of Sindbis/eastern equine encephalitis chimeric viruses in plaque reduction neutralization tests for arboviral disease diagnostics. Clin Vaccine Immunol. (9):1486-91. doi: 10.1128/CVI.05129-11.

5. Quiroz E, Aguilar P v., Cisneros J, Tesh RB, Weaver SC. 2009. Venezuelan equine encephalitis in Panama: Fatal endemic disease and genetic diversity of etiologic viral strains. PLoS Negl Trop Dis;3(6):e472. doi: 10.1371/journal.pntd.0000472.

6. Claro IM, Romano CM, Candido D da S, de Lima EL, Lindoso JAL, Ramundo MS, Moreira FRR, Barra LAC, Borges LMS, Medeiros LA, Tomishige MYS, Moutinho T, da Silva AJD, Rodrigues CCM, de Azevedo LCF, Villas-Boas LS, da Silva CAM, Coletti TM, Manuli ER, O’toole A, Quick J, Loman N, Rambaut A, Faria NR, Figueiredo-Mello C, Sabino EC. 2022. Shotgun metagenomic sequencing of the first case of monkeypox virus in Brazil, 2022. Rev Inst Med Trop Sao Paulo. 2022 Jun 24;64:e48. doi: 10.1590/S1678-9946202264048.

7. Li H. 2018. Minimap2: Pairwise alignment for nucleotide sequences. Bioinformatics. 2018 Sep 15;34(18):3094-3100. doi: 10.1093/bioinformatics/bty191.

8. Milne I, Stephen G, Bayer M, Cock PJA, Pritchard L, Cardle L, Shawand PD, Marshall D. 2013. Using tablet for visual exploration of second-generation sequencing data. Brief Bioinform: 14(2):193-202. doi: 10.1093/bib/bbs012.
